# Opinion: On the reasons for underutilization of ECT in individuals with intellectual and developmental disability

**DOI:** 10.3389/fpsyt.2026.1810364

**Published:** 2026-07-03

**Authors:** Melanie Johnston, Katie Brooker, Catherine Franklin

**Affiliations:** Queensland Centre of Excellence in Intellectual Disability and Autism Health, Mater Research Institute-University of Queensland (UQ), The University of Queensland, QLD, Brisbane, Australia

**Keywords:** autism, developmental disability, electroconvulsive therapy, healthcare equity, intellectual disability

## 1 Introduction

Individuals with intellectual and developmental disabilities, including autistic individuals, experience profound and persistent inequities accessing healthcare (1, 2). These inequities span preventive care, physical health, and mental health services, and are evident across countries, health systems, and funding models (3). Within this broader landscape of structural disadvantage, access to electroconvulsive therapy (ECT) represents a particularly stark example of inequitable care. ECT is a well-established, guideline-supported intervention delivered across public and private hospital systems internationally and remains one of the most effective treatments for severe, treatment-refractory mood and affective disorders, psychotic disorders, and catatonia (4–6).

Yet paradoxically, individuals with intellectual and developmental disabilities are more likely than the general population to experience the very conditions for which ECT is indicated, such as catatonia, severe affective illness, and psychosis (7), while receiving ECT at substantially lower rates (8, 9). Despite limited representation in clinical research, available evidence consistently demonstrates that ECT is comparably safe and effective in this population compared to general adult cohorts (9, 10).

The underutilization of ECT therefore cannot be explained by lack of clinical need, or by insufficient evidence of its efficacy and tolerability. Instead, it reflects the combined effects of historical mistrust, entrenched stigma, clinician discomfort, defensive medicine, structural inequities, and health-economic pressures that disproportionately disadvantage people with disability. For contemporary psychiatry, confronting these forces is essential. Ensuring equitable access to ECT is not simply a matter of clinical optimization but is central to the ethical provision of disability-inclusive mental health care.

## 2 Body

### 2.1 The legacy of twentieth century psychiatry: inclusion versus protection of persons with a disability

Psychiatry continues to practice in the shadow of its twentieth-century history. The profession’s involvement in coercive institutionalisation, experimental psychosurgery, and eugenic practices has left an enduring legacy of mistrust, especially among individuals with disability and their advocates. Individuals with intellectual and developmental disabilities were disproportionately subjected to non-consensual interventions, and psychiatric treatments were often administered as instruments of social control rather than therapeutic care (11).

ECT is unavoidably entangled in this history. Early forms of ECT were delivered without anaesthesia or paralytic agents, often in institutional settings and frequently without meaningful consent. These practices, now unequivocally recognised as unethical, continue to shape public and professional perceptions of ECT decades later. Popular media portrayals reinforce this narrative, depicting ECT as punitive, violent, or dehumanising, while rarely acknowledging the profound transformation in safety, tolerability, and ethical governance that characterises contemporary practice.

For individuals with intellectual and developmental disabilities, this history carries particular weight. Contemporary disability rights frameworks emphasise autonomy, supported decision-making, and least restrictive practice. Within this context, any intervention perceived as coercive or invasive is scrutinised intensely. Although this scrutiny is important and appropriate, when influenced by lay opinion and historical narratives, it may inadvertently render ECT uniquely suspect when offered to people with disability, even when it represents the safest and most effective option available for the clinical circumstances.

Modern ECT bears little resemblance to its historical antecedents. It is administered under general anaesthesia with physiological monitoring, and stringent consent processes and legislative oversight. Yet the persistence of historical narratives means that ECT remains symbolically burdened in a way few other psychiatric treatments are. For individuals with intellectual and developmental disabilities whose voices have historically been marginalized, this burden can translate into overly-cautious refusal rather than balanced ethical consideration.

Despite major advances in psychiatric ethics and practice, the realities of modern ECT remain largely invisible to the public. ECT remains stigmatized and stigmatizing. Patients with intellectual and developmental disabilities and their families and support people do not commonly share their experiences, which limits opportunities to counter misinformation with lived experience. In contrast, dramatic portrayals of ECT circulate freely in modern media, amplified by social media and algorithm-driven content that preferences sensational content over accuracy.

In the absence of accessible and trustworthy information, families and carers of individuals with intellectual and developmental disabilities are often left navigating conflicting narratives, at a time of heightened vulnerability. This informational asymmetry disproportionately affects individuals with intellectual and developmental disabilities, who may rely heavily on supported or substitute decision-makers and whose access to nuanced explanations may be constrained by communication barriers.

The result is a form of ethical paralysis: clinicians fear being perceived as coercive, families fear causing harm, and systems default to inaction. Yet in the context of severe psychiatric illness, inaction is not neutral. Untreated catatonia, refractory depression, or psychotic illness all carry substantial morbidity and mortality, including loss of function, loss of independence, prolonged hospitalisation, medical complications, and death (12, 13). When ECT is withheld because of reputational anxiety rather than clinical judgment, the patient bears the cost.

### 2.2 Therapeutic nihilism, confidence gaps, and defensive medicine

A further driver of ECT underutilisation is therapeutic nihilism— in this context manifesting as the implicit belief that people with intellectual and developmental disabilities are less likely to benefit from, or tolerate, psychiatric interventions; or that treatment gains will be limited by the baseline presence of disability. This belief persists despite evidence that individuals with intellectual and developmental disabilities can experience substantial improvements in mood, behaviour, and quality of life when comorbid mental illness is treated effectively and accessibly (14).

Psychiatrists frequently report low confidence in assessing and treating individuals with intellectual and developmental disabilities, particularly when presentations are complex or atypical (15). Diagnostic overshadowing, communication differences, and behavioural manifestations of distress can obscure classical symptom profiles. In this context, clinicians may hesitate to escalate to ECT, viewing diagnostic uncertainty as a contraindication rather than as an indicator of illness severity that should prompt sub-specialist input.

These confidence gaps are compounded by limited exposure to developmental psychiatry during training. Many psychiatrists complete their education with minimal experience treating individuals with intellectual and developmental disabilities, and even less exposure to the use of ECT in these populations. Unsurprisingly, clinicians are less inclined to recommend a treatment they have never seen used.

Importantly, such reluctance is rarely framed explicitly as discrimination. Instead, it is justified through appeals to caution, protection, or uncertainty. However, when caution is applied selectively to one group, it becomes a mechanism of inequity. Individuals without disability are routinely offered ECT on the basis of clinical severity and risk; individuals with intellectual and developmental disabilities are often required to meet vague additional, informal thresholds of reassurance.

Concerns about decision-making capacity are frequently cited as barriers to ECT access for individuals with intellectual and developmental disabilities. While capacity assessment is a core ethical requirement, it is often misunderstood or inconsistently applied. Capacity is decision-specific and support-dependent; it is not negated by the presence of intellectual and developmental disabilities. Furthermore, many individuals requiring ECT will lack capacity to consent at the point of needing treatment: this is not a unique issue to intellectual and developmental disabilities, and alternative consent processes are, or should be, standard.

In practice, individuals with intellectual and developmental disabilities are more likely to be deemed decisionally impaired or incapable, and substitute decision-making pathways are invoked earlier and more readily. This, heightens institutional anxiety about legal risk and reputational harm, particularly given the historic context. ECT, already symbolically burdened, becomes a treatment of last resort even when legally authorised and clinically indicated.

Ironically, this heightened protectiveness may undermine the very rights it seeks to defend. Prolonged suffering, repeated restraint, polypharmacy, and extended involuntary hospitalisation may all be tolerated in the name of avoiding ECT, despite carrying their own significant ethical and physical risks. A disability-rights-informed approach must consider whether withholding effective treatment constitutes a greater harm than its careful, supported, and ethically governed provision.

### 2.3 Health economics, resource distribution, equity, and equality

Beyond attitudinal barriers, structural factors play a significant role in underutilization of ECT. ECT services are resource-intensive and often operate at or near capacity, with finite treatment slots available per week. Within many health systems, resource allocation follows a utilitarian approach in which priority is given to individuals perceived as having the greatest likelihood of rapid improvement and timely discharge.

Individuals with intellectual and developmental disabilities frequently present with conditions such as autism-associated catatonia or severe affective illness that may require prolonged ECT courses or ongoing maintenance treatment (12). From a service management perspective, such cases are often viewed as open-ended and resource-heavy. As a result, implicit pressures may arise to reserve ECT for patients with less comorbidities or complexities, effectively disadvantaging those with intellectual and developmental disabilities and multiple additional conditions adding to complexity.

This pattern reflects an equality-based rather than equity-based model of care. Treating everyone “the same” within a constrained system inevitably benefits those who align with standard pathways. Equity requires recognising that some individuals need more intensive or longer-term intervention to achieve comparable outcomes. When systems fail to accommodate this, they perpetuate structural discrimination against individuals with intellectual and developmental disabilities.

The underutilisation of ECT in individuals with intellectual and developmental disabilities should therefore be understood not merely as a clinical pattern, but as a manifestation of systemic inequity. ECT is among the most effective interventions in psychiatry, with rapid onset and robust efficacy in conditions associated with high morbidity and mortality (16). Denying access to such a treatment on the basis of disability, whether explicitly or implicitly, contravenes principles of justice and non-discrimination.

Reframing ECT as an equity issue requires a shift in professional culture. It demands acknowledgement of historical harms without allowing them to justify present-day neglect. It requires investment in clinician training, specialist consultation pathways, and accessible, supported consent processes. Crucially, it also requires transparent engagement with disability communities to rebuild trust through openness rather than avoidance and defensiveness.

### 2.4 Acknowledging the risks and limitations of ECT

It is the case that individuals with intellectual disability may have increased rates of medical comorbidity, epilepsy, anatomical differences, sensory sensitivities, and neurodevelopmental conditions that can complicate assessment, consent processes, anaesthetic management, and monitoring of adverse effects (17). The administration of ECT carries risks, including the risk of anaesthetic, of cognitive side effects, and from the logistical burden of treatment for individuals who may not tolerate clinical environments well, making administration particularly distressing. Cognitive adverse effects, including transient confusion and memory disturbance, may be more difficult to detect due to pre-existing cognitive impairment and challenges establishing baseline functioning, and may also cause significant distress.

It is certainly the case that ECT may be contraindicated for some individuals with co-occurring medical conditions or be deemed to have risks outweighing the benefit. But this clinical dilemma exists for individuals with and without disability, all of which should be considered during informed consent processes. There are individuals both with and without intellectual and developmental disability for whom the administration of ECT is either not safe, not feasible, or otherwise not within their best interests; which needs consideration on a case-by-case basis.

## 3 Discussion

ECT remains underutilised among individuals with intellectual and developmental disabilities despite clear clinical indications, an established evidence base, and decades of progress in ethical psychiatric practice. This disparity cannot be adequately explained by concerns about safety, efficacy, or consent alone. Rather, it reflects the cumulative effect of historical mistrust, persistent stigma, therapeutic nihilism, clinician confidence gaps, and structural constraints within health systems that continue to disadvantage people with disability.

Importantly, the ethical discomfort surrounding ECT in this population has produced a pattern of risk aversion that is itself ethically consequential. When effective treatment is withheld, even by clinicians with protective intent, individuals with intellectual and developmental disabilities may experience prolonged suffering, repeated restraint, escalating polypharmacy, medical complications, loss of functional skills, and extended involuntary hospitalisation. These outcomes are rarely framed as ethical failures, yet they frequently carry greater cumulative harm than the careful, rights-based use of ECT. There are risks involved in accessing ECT, however as in all clinical decisions, they need to be applied to the individual and weighed up appropriately with informed consent processes. Underutilisation of ECT, in the sense described here, represents not protection, but systemic neglect.

Addressing this inequity requires more than incremental change. Psychiatry must move beyond defensive practice shaped by historical shame and public misunderstanding and instead adopt a proactive, equity-oriented approach. This includes improving training and exposure to intellectual and developmental disability psychiatry and ECT, strengthening access to specialist consultation, embedding supported decision-making into consent processes, and ensuring that service design and funding models do not systematically exclude individuals who require longer or more complex treatment courses.

Equally critical is transparent engagement with people with intellectual and developmental disabilities, families, and advocacy communities in advisory, research, and clinical co-design roles. Rebuilding trust will not be achieved by silence or avoidance, but by openly acknowledging past harms while clearly articulating the realities of contemporary ECT practice.

Ultimately, the question is not whether ECT is ethically permissible in people with intellectual and developmental disabilities. The question is whether psychiatry can justify a system in which the presence of intellectual and developmental disabilities so reliably predict exclusion from highly effective care. If equity is taken seriously as a core principle of modern mental health care, then improving equitable access to ECT for individuals with intellectual and developmental disabilities is a moral, clinical, and professional obligation.
